# Supplementary material for: Structured Routine Use of Styletubation for Oro-Tracheal Intubation in Obese Patients Undergoing Bariatric Surgeries—A Case Series Report
Source: Healthcare (Basel). 2024 Jul 15;12(14):1404. doi: 10.3390/healthcare12141404 (PMC11276324; doi:10.3390/healthcare12141404)
Supplement: Supplementary file 1 [file healthcare-12-01404-s001.zip › healthcare-3075282-supplementary.pdf]

Supplementary Material

Supplementary	Access Link
Video S1	https://drive.google.com/file/d/1zvDJRK03F2OuIXdQCFUcQ4UpqpkFYtYJ/view
Video S2	https://drive.google.com/file/d/19bG9Vd3dCCm8p3QO360m1wBz-_QvvnWO/view
Video S3	https://drive.google.com/file/d/1QG-3rO9Lhmn97Jj-Kmq5g_h6d-_7nwLg/view
Video S4	https://drive.google.com/file/d/1eWoHQ3DJ1KhoUgZs6I8q37hw7bfIeFfM/view
Video S5	https://drive.google.com/file/d/18ITwteogAbf3cYNtie9BEAOhGpH8vA3g/view
Video S6	https://drive.google.com/file/d/1dB8ab1SzSvOGXUNkU4VwD1nyC3KPHU_5/view
Video S7	https://drive.google.com/file/d/1FS0cMqxVMFEJ-e30tBap4rHAzPJlfhhR/view
Video S8	https://drive.google.com/file/d/1rbHh0bOs37yeO3D5SVUF5Yx3VBXJPWAu/view
Video S9	https://drive.google.com/file/d/1U7MdLfJPuo3ba0HMsvCQDvDkv6V5jETM/view
Video S10	https://drive.google.com/file/d/1ju_dJoMh48B-DkdcGphHoik94dKO-3xE/view
Video S11	https://drive.google.com/file/d/1ZK5vw1MV4XklGtWJq9wMIV1z0GPD_Mp1/view
Video S12	https://drive.google.com/file/d/1uuj4YtwoaMuOk8PjXArPVQQBIFkwN3bU/view
Video S13	https://drive.google.com/file/d/1I1CnHPNKcfru4asTuF5tGCRrSoqwdwgk/view
Video S14	https://drive.google.com/file/d/168UN8t9PnHJmkL-ieGVFASOutBnMNy9-/view
Video S15	https://drive.google.com/file/d/13mOKrdlsM0WXnplwuRdNaybC8dHCFees/view
Video S16	https://drive.google.com/file/d/1_U--SxbKmUDX3UBThLqe3MSzJu_5b7XB/view
Video S17	https://drive.google.com/file/d/1Nr6eFfNH8_RI6bNJxMf-QWeATUu8cuXm/view
Video S18	https://drive.google.com/file/d/1rMT0HFAJM1goPHybL3cnUWSo1yyGuS1Y/view
Video S19	https://drive.google.com/file/d/1JzBuXjASFBjfcF4dQ3QOnRkQqWmsLkoD/view
